# Sex-divergent trajectories of hippocampal and cortical NMDA receptor density across the Alzheimer’s disease continuum

**DOI:** 10.64898/2026.08.05.743051

**Published:** 2026-08-06

**Authors:** Maricedes Acosta-Martínez, Vanessa Carter, Aviram Nessim, Sarah Murphy, Jasbeer Dhawan, Thomas G. Beach, Geidy E. Serrano, Erin E. Sundermann, Anat Biegon

## Abstract

While loss of NMDA receptors (NMDARs) is associated with Alzheimer’s disease (AD) severity, the effect of sex or the relationship between regional NMDAR density and antemortem cognitive status across the AD spectrum has not been examined. We performed quantitative *in vitro* autoradiography of hippocampus, entorhinal cortex (EC), and parietal cortex using NMDAR and tau radioligands. Relationships between regional NMDAR density and cognitive status assessed by the Mini Mental State Exam (MMSE), and between NMDAR and tau density, were examined by bivariate correlations. In both sexes, the largest AD-related decreases in NMDAR density were observed in the CA1 field. However, there was a significant diagnosis by sex interaction driven by sex-specific changes in the mild cognitive impairment (MCI) stage, with lower NMDAR density in MCI women, but not MCI men relative to same-sex controls. Within diagnosis analyses revealed positive correlations between NMDAR density and MMSE scores and significant negative correlations between EC NMDAR and tau density, which was significant only in AD men. Our data show that changes in hippocampal NMDAR density across the AD continuum are modulated by sex and may contribute to the known sex differences in the clinical trajectory of the disease.

## Full Text Availability

The license terms selected by the author(s) for this preprint version do not permit archiving in PMC. The full text is available from the preprint server.

